# Antidiarrhoeal Activity of Leaves of *Melastoma malabathricum* Linn.

**DOI:** 10.4103/0250-474X.59556

**Published:** 2009

**Authors:** J. A. J. Sunilson, K. Anandarajagopal, A. V. A. G. Kumari, S. Mohan

**Affiliations:** School of Pharmacy, Masterskill University College Health Sciences, G-8, Jalan Kemacahaya 11, Taman Kemacahaya, Batu-9, Cheras 43200, Selangor, Malaysia

**Keywords:** Antidiarrhoeal activity, castrol-induced diarrhea, *Melastoma malabathricum*, water extract of the leaf

## Abstract

The antidiarrhoeal effect of the water extract of *Melastoma malabathricum* Linn. (Melastomataceae) leaves were investigated by employing four experimental models of diarrhea in Swiss mice. *Melastoma malabathricum* water extract treated mice showed significant reduction in the fecal output and protected them from castor oil-induced diarrhoea. The extract also reduced the intestinal fluid secretion induced by magnesium sulphate and gastrointestinal motility after charcoal meal administration in the mice. No mortality and visible signs of general weakness was observed in the mice following the test extract administration up to 2000 mg/kg dose.

Diarrhea, which could be infectious or non-infectious, is one of the principal causes of death, particularly in the malnourished infants[1]. In order to combat the problems of diarrhoea globally, the World Health Organization in its Diarrhoeal Disease Control programme has given a special emphasis on the use of traditional folklore medicines in the control and management of diarrhea[2]. *Melastoma malabathricum* Linn. (Melastomataceae) is a erect shrub or small tree 1.5 to 5 m tall, found more or less everywhere throughout Malaysia[3]. It's commonly called Straits Rhododendron and locally known as *Senduduk*[4,5]. It was traditionally used to treat diarrhoea, dysentery, leucorrhoea, hemorrhoids, wounds, infection during confinement, toothache, flatulence, sore legs and thrush and also it is used by the Jah hut people in Malaysia to cure diarrhea[6]. Chemical investigation of the plant showed the presence of naringenin, kaempferol, kaempferol-3-O-glucoside and kaempferol-3-O-(2”, 6”-di-O-P-trans-coumarouyl)glucoside[7].

The aim of the present study was to evaluate the possible antidiarrhoeal activity of the leaf extract of *Melastoma malabathricum*, in order to scientifically evaluate the claimed biological activities of this plant. The leaves of *M. malabathricum* were collected from Taman Kemacahaya, Selangor, Malaysia in the month of November 2007. The plant was identified at the Department of Botany, Science University of Malaysia, Penang, Malaysia. A voucher specimen (No. MSCNH/M-1(8), 2007) was deposited in our departmental herbarium. The leaves were shade dried, powdered and sieved through 40 mesh sieve.

Swiss mice (18-20 g) kept at the Laboratory Animal House of Masterskill University College of Health Sciences, Cheras, Malaysia were used. They were kept in well cross ventilated room at 27±2°, for 1 w before and during the experiments. Animals were provided with commercial rodent pellet diet and water *ad libitum*. The study protocol was approved by the animal ethical committee, Masterskill College of Nursing and Health, Malaysia. All the experiments were performed according to current guidelines for the care of the laboratory animals and the ethical guidelines. The protocol was approved by IAEC No: (MSCNS/F-209(f)/014) dated on 06/11/07. The standard orogastric cannula was used for oral drug administration

The powdered plant materials 500 g were macerated in 1.5 l distilled water for 48 h[8]. After filtration, the filtrate was concentrated and dried under reduced pressure. The water extract was dark-green and semisolid. The yield was 23.41% (w/w). The extract was stored in desiccators until use.

The acute toxicity studies described by Miller *et al*.[9] was employed in the determination of the LD_50_. Water extract was administered orally at a dose of 62.5, 125, 250, 500, 1000 and 2000 mg/kg to a group of 5 animals each. The general signs and symptoms of toxicity, intake of food and water and mortality were recorded for 48 h.

The antidiarrhoeal efficacy of *M. malabathricum* leaves water extract (MMMW) was assessed by four different experimental models. Five groups of animals were housed in separate cages having paper placed below for collection of fecal matters. Group 1 (control) received distilled water; groups 2-4 were treated with MMMW at 100, 200 and 500 mg/kg, p.o., respectively. The 5th group received 0.5 ml of 5 mg/kg loperamide. The fecal material collected for 12 h post treatment was dried in an incubator and weighed. The percentage reduction in the fecal output was determined[10].

In overnight fasted male mice, diarrhoea was induced by oral administration of castor oil (0.5 ml/mouse, p.o.). The animals were randomized into five groups of 5 mice each. Group 1 served as control and received distilled water; Groups 2-4 were given orally the MMMW extract (100, 200 and 500 mg/kg) 1 h prior to castor oil administration. The remaining group 5 received 5 mg/kg of loperamide as standard. The percentage protection from diarrhoeal droppings was calculated[11].

In enteropooling assay method, five groups of 5 animals each fasted overnight were used. Group 1 was used as control, while groups 2-4 received the MMMW extract (100, 200 and 500 mg/kg p.o., respectively). The last group 5 was given the standard drug, loperamide (5 mg/kg). One hour later, all groups were given the diarrhoeal agent (0.5 ml / mouse of a 10% aq MgSO_4_, orally). They were killed 30 min later and the small intestines were collected and weighed to find out the accumulation of intestinal fluid secretion evoked by MgSO4[12].

Gastrointestinal transit test was performed using five groups of 5 animals each. All the animals were fasted over night. The test extract was given orally to group 2-4 (100, 200 and 500 mg/kg, respectively), while group 1 used as control; the 5th group received loperamide (5 mg/kg) as a standard. Five minutes later, 0.5 ml of 3% charcoal suspended with tragacanth powder was administered orally to each mouse. All the mice were killed by cervical dislocation 30 min later and the distance travelled by the charcoal plug from pylorus to caecum was determined and expressed as a percentage of the total length of the small intestine[13,14]. The significance of difference between the means was determined by the Student's ‘*t*’ test and the results were regarded as significant when *P*<0.05.

The acute toxicity study showed that oral administration of water extract of *M. malabathricum* leaves to the mice up to 2000 mg/kg dose neither showed mortality nor any visible clinical signs of general weakness in the animals.

The results revealed that MMMW tested at the concentration of 100, 200 and 500 mg/kg reduced the fecal output of the mice by 30.13%, 39.73% and 43.19%, respectively, while the reduction in the fecal output by loperamide (5 mg/kg) was noted to be 57.39% when compared to the control group Table 1.

**TABLE 1:** EFFECT OF WATER EXTRACT OF *M. MALABATHRICUM* LEAVES ON FAECAL OUTPUT IN MALE ALBINO MICE

Experimental group	Dose (mg/kg, p.o.)	Dried fecal output per 100 g of mice	% Reduction in fecal output
Control	-	0.521±0.083	0.00
MMMW	100	0.364±0.012*	30.13
MMMW	200	0.314±0.046*	39.73
MMMW	500	0.296±0.023*	43.19
Loperamide	5	0.222±0.015*	57.39

* Values are mean±SEM. P<0.05, significantly different from control group, Student's t-test (n=5 per group). MMMW is *Melastoma malabathricum* water extract

The MMMW extract (100, 200 and 500 mg/kg) protected the mice against castor oil induced diarrhoeal droppings by 60–80% where as the protection was noticed to be 100 % in the case of treatment by 5 mg/kg of loperamide Table 2.

**TABLE 2:** EFFECT OF WATER EXTRACT OF *M. MALABATHRICUM* LEAVES ON CASTOR OIL-INDUCED DIARRHOEA IN MICE

Experimental group	Dose (mg/kg, p.o.)	No. of mice with diarrhoeal droppings within 4 h	Protection (%)
Control	-	5/5	0
MMMW	100	2/5	60
MMMW	200	1/5	80
MMMW	500	1/5	80
Loperamide	5	0/5	100

*Average of 5 animals. MMMW is *Melastoma malabathricum* water extract

The extract reduced the intestinal fluid secretion induced by MgSO_4_, in a dose dependant fashion Table 3. The reduction in the intestinal fluid secretion at 500 mg/kg of MMMW extract treatment was found to be almost comparable with that of treatment by 5 mg/kg dose of loperamide.

**TABLE 3:** EFFECT OF WATER EXTRACT OF *M. MALABATHRICUM* LEAVES ON ENTEROPOOLING ASSAY IN MICE

Experimental group	Dose (mg/kg, p.o.)	No. of mice in the group	Wt. of the small intestine per 100 g of mice
Control	-	5	9.362±0.518
MMMW	100	5	8.413±0.431*
MMMW	200	5	7.620±0.469*
MMMW	500	5	7.314±0.261*
Loperamide	5	5	6.416±0.514*

* Values are mean±SEM. P<0.05, significantly different from control group, Student's t-test (n=5 per group). MMMW is *Melastoma malabathricum* water extract

The MMMW extract (100, 200 and 500 mg/kg) inhibited the small intestinal motility of the charcoal marker in mice by 8.10–32.97% whereas the inhibition was noted be 57.42% in the case of loperamide Table 4.

**TABLE 4:** EFFECT OF WATER EXTRACT OF *M. MALABATHRICUM* LEAVES ON GASTROINTESTINAL TRANSIT IN MICE

Experimental group	Dose (mg/kg, p.o.)	Distance travelled by marker as % of total length of small intestine	% Inhibition
Control	-	69.24±5.03	0.00
MMMW	100	63.63±3.71*	8.10
MMMW	200	51.82±4.11*	25.15
MMMW	500	46.41±3.25*	32.97
Loperamide	5	29.48±2.69*	57.42

* Values are mean±SEM. P<0.05, significantly different from control group, Student's t-test (n=5 per group). MMMW is *Melastoma malabathricum* water extract

In traditional medicine system, many plants or herbs are claimed to have antidiarrhoeal efficacy without any scientific basis. The aim of the present study was to evaluate the antidiarrhoeal effects of the leaves of *M.malabathricum*, which are consumed very commonly by the Jah hut people in Malaysia, to treat the diarrhea. In establishing the pharmacological evaluation of a potential antidiarrhoeal agent, the inhibition of experimentally induced diarrhea, reduction in the fecal output and gastrointestinal motility tests have remained the most common parameters in several studies[15–17]. The present study revealed that the water extract of *M. malabathricum* leaves inhibited significantly the frequency of defecation and reduced greatly the wetness of the fecal excretion like the standard antidiarrhoeal agent, loperamide. The therapeutic effect of loperamide is believed to be due to its antimotility and antisecretory properties[18]. The extract also significantly protected the mice from diarrhoeal droppings evoked by castor oil administration. Drugs affecting motility, frequency and consistence of diarrhea also affect secretion[19]. The intraluminal fluid accumulation induced by castor oil was blocked by the test extract in dose-related manner. Further, the experiments carried out on the gastrointestinal tract motility after charcoal meal administration also showed a reduction in the propulsive movement of small intestine after pre-treatment with the extract of *M. malabathricum.* Intestinal fluid secretion has been analyzed by enteropooling assay in mice, evoked by MgSO_4_ (a standard laxative agent).

In conclusion, the results of this study seem to provide a support for the use of *M. malabathricum* leaves as antidiarrhoeal agent in the traditional medicine system of the Jah hut people in Malaysia. Further study, however, is necessary to isolate and identify the active ingredients and their precise mechanism of action.
